# Ethnomedicinal values of *Boerhaavia diffusa* L. as a panacea against multiple human ailments: a state of art review

**DOI:** 10.3389/fchem.2023.1297300

**Published:** 2023-11-14

**Authors:** Sarita Das, Puneet K. Singh, Shaikh Ameeruddin, Birendra Kumar Bindhani, Wajdi J. Obaidullah, Ahmad J. Obaidullah, Snehasish Mishra, Ranjan K. Mohapatra

**Affiliations:** ^1^ Microbiology Laboratory, Department of Botany, Berhampur University, Berhampur, Odisha, India; ^2^ Bioenergy Lab, School of Biotechnology, Campus-11, KIIT Deemed-to-be-University, Bhubaneswar, Odisha, India; ^3^ School of Biotechnology, Campus-11, KIIT Deemed-to-be-University, Bhubaneswar, Odisha, India; ^4^ General Department of Medical Services, Ministry of Interior, Riyadh, Saudi Arabia; ^5^ Department of Pharmaceutical Chemistry, College of Pharmacy, King Saud University, Riyadh, Saudi Arabia; ^6^ Department of Chemistry, Government College of Engineering, Keonjhar, Odisha, India

**Keywords:** antidiabetic, antimicrobial, anticancer, *Boerhaavia diffusa*, flavonoids, phenolics, punarnavine, bioactivity

## Abstract

**Ethnopharmacological relevance:** Therapeutic botanicals (plants and derivatives) are in use since antiquity for various health ailments. The ethnic community is the repository of the information, the multifactorial therapeutic applications of which may often need scientific validation. The spreading hogweed or *Boerhaavia diffusa* L., also known as Punarnava, is a reassuring medicinal herb with diverse pharmacological benefits. It is used in Ayurveda in Asia and Africa as a rejuvenator or “Rasayan” for its excellent antiaging and antioxidant properties.

**Aim:** The study aimed at compiling the state-of-art knowledge of the medicinal benefits of *Boerhaavia diffusa* L. and unraveling the unexplored commercially useful bioactive constituents by establishing their possible pharmacological benefits.

**Methods:** The data from published literature, confined to pharmacological manifestations of various phytocomponents of *Boerhaavia diffusa* L. or its parts like root, leaf and stem were extracted from scientific databases, Google, Science Direct, PubMed, etc. using its antifungal, antibacterial, anticancer, anti-inflammatory, antidiabetic, hepatoprotective, cardioprotective, renoprotective, antifertility benefits and molecular docking study as search strings and keywords. Further, the reported *in silico* studies for bioactivity and bioavailability are detailed.

**Results:** The botanicals possess numerous bioactive compounds, the most widely reported ones being phenolic (punarnavoside, trans-caftaric acid, boerhavic acid), rotenoid (boeravinones A-J), flavonoid (borhaavone, quercetin, kaempferol), isoflavonoid (2′-O-methyl abronisoflavone), alkaloid (punarnavine), steroid (boerhavisterol, *β*-Ecdysone), anthracenes and lignans (liriodendrin, syringaresinol mono-*β*-*D*-glucoside). Some of the reported reassuring benefits of their purified forms or even the crude extracts are antidiabetic, antimicrobial, anticancer, antioxidant, anti-inflammatory, hepatoprotective, renoprotective, cardioprotective, antifertility, etc.

**Conclusion:** The article provides an extensive study on such pharmacological utility to support the ethnomedicinal use of *Boerhaavia diffusa* L. and propose possible mechanism of the various bioactive compounds in optimising metabolic dysfunctions, healing and protecting vital body organs, often related to the magnificent antioxidant property of this ayurvedic panacea. Further, establishing specific roles of its yet-to-explore bioactive constituents for diverse pharmacological applications is suggested.

## Graphical Abstract

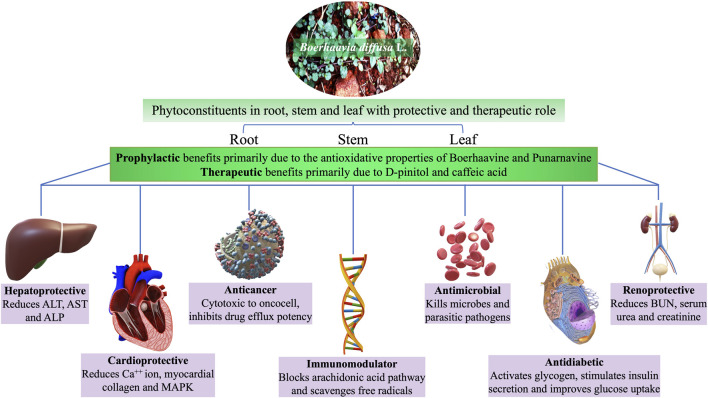

## Highlights

1. Popular among ethnic communities as a leafy vegetable, every plant part has medicinal value
2. Plant parts have protective and therapeutic values especially towards critical internal organs
3. Being herbal derivatives, majority of the bioactive compounds are evidently less cytotoxic
4. Few derived bioactive compounds are commercially exploited; few others are unexplored yet
5. The prophylactic and therapeutic properties of bioactive extracts are detailed bioinformatically
6. Exploitation of the antimicrobial properties could protect humans against infectious diseases

## Introduction

**FIGURE 1:**
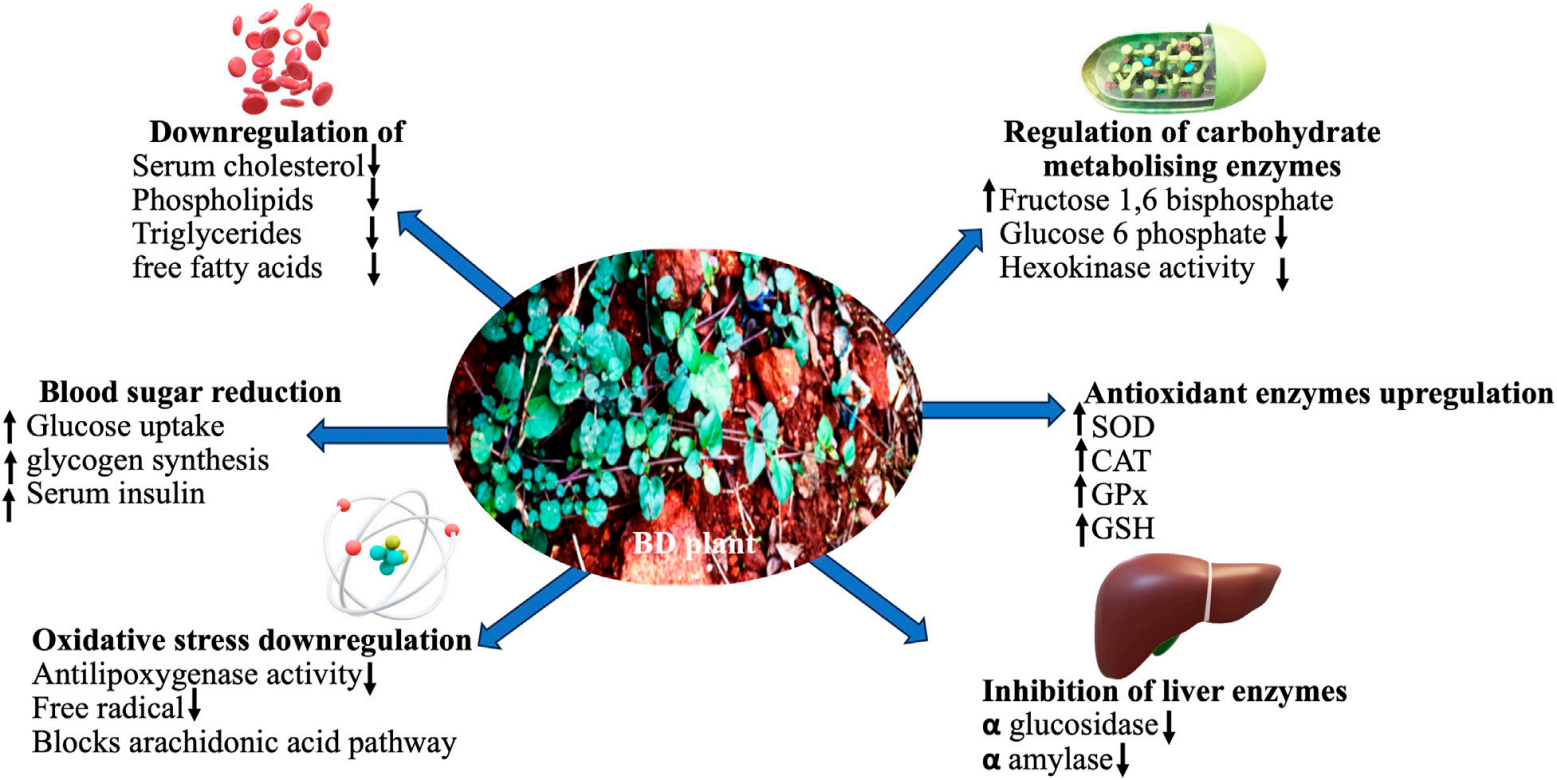
Mechanisms of the manifestation of antidiabetic property of BD phytocompounds.

*Boerhaavia diffusa* L. (BD) of family Nyctaginaceae, commonly known as spreading hogweed, is a perennial, prostrate herb with pink flowers and sticky fruits (Figure 1). Popularly called Punarnava, it means renewer or rejuvenator of body because of its anti-aging property (Wahi et al., 1997). Its old root stock remains dormant during summer and regenerates during the rain. The various parts of Punarnava are rich in diverse bioactive compounds and are extensively used as a “rasayan” for its exceptional properties like immunity boosting, reestablishing youthfulness, and strengthening the body and mind (Samhita and Varanasi, 1998). Oxidative stress (due to an imbalance between the reactive oxygen species or oxidants production and their utilisation) often leads to diseases like dementia, Alzheimer’s disease, Parkinson’s disease, heart attack, myocardial infarction and other gerontological ailments (Demopoulos et al., 1980; Cadet, 1998). Promising antioxidant property of the BD parts makes it special for pharmacological applications (Das et al., 2022). Both the root and the shoot systems of this wonder plant are rich primarily in phenolics (punarnavoside, trans-caftaric acid and boerhavic acid), rotenoids (boeravinones A-J), flavonoids (borhaavone**,** quercetin, kaempferol), isoflavonoids (2′-O-methyl abronisoflavone), alkaloids (punarnavine), steroids (boerhavisterol, *β*-Ecdysone), anthracenes and lignans (liriodendrin, syringaresinol mono-*β*-*D*-glucoside) and many fatty acids and proteins contributing to its incredible biotherapeutic potential (Gaur et al., 2022). Its antidiabetic, antimicrobial and other similar bioactivities of BD is extensively studied, reviewed and are reported often.

Although, historically and ethnomedicinally the plant has been associated among especially the Asian and African ethnic population both as a food as well as an ayurvedic intervention, however the transition into the modern civilisation and the medical sciences is yet to realise its potential. The wellbeing benefits of BD are so much more everencompassing and reassuring that, considering it as a panacea against an array of modern day diseases shall not be an overstatement. In light of this, extensive research and both vertical (deeper insights into the *heather to* unknown medicinal benefits) as well as horizontal (spread of the medicinal benefits in the global population across ethnicity and race defying the geographical barriers) understandings shall benefit the humanity in general and the ethnic population in particular.

This article attempts to compile the advances on the therapeutic role of BD for multiple human health benefits including in metabolic disorders, vital organ protection, antimicrobial, anticancer, anti-inflammatory and healing properties against various diseases especially in the last decade (2013–2023), to validate and support its ethnomedicinal utility. It attempts also to reorients and synthesise all its pharmacological aspects, the possible relationship between phytocompounds, the bioactivities, and the probable mechanism of action in blood sugar management. BD is an incredible herbal remedy containing an array of pharmaceutically active chemical components.

BD has free radicals scavenging and glutathione content increasing antioxidants that help treat hepatitis and hepatic cirrhosis. BD leaf is a source of significant quantity of flavonoid glycoside moiety with high eupalitin-3-O-D-galactopyranoside (yellow powder crystal) yield. Silymarin, a flavonoid glycoside structurally similar to eupalitin-3-O-D-galactopyranoside was the only known natural stuff used for hepatoprotection till late. Human hepatoma cell lines are proposed as an alternative to human hepatocytes for *in vitro* modeling of normal liver cells. HepG2 hepatoma cell line is widely used in liver function, metabolism and drug toxicity research. The biochemical and morphological properties of these cells being similar to normal hepatocytes, they are used in studies that determine the hepatoprotective properties of medicinal plants.

## Materials and methods

Relevant research and review articles were considered, and the mentioned aspects were collected and compiled. Extensive literature search on the medicinal, antimicrobial and pharmacological properties of the whole plant or the parts of BD was conducted using specialised and dedicated search engines and websites like Google Scholar, PubMed, ScienceDirect, EBSCO, Sci-Hub, SciFinder, etc. Major thrust was on the literature that covered the antibacterial, antifungal, anticancer, anti-inflammatory, hepatoprotective, cardioprotective, renoprotective, antidiabetic, antifertility characteristics of BD or its parts (like root, leave, stem, etc.) and molecular docking study as keywords.

### Ethnomedicinal usage

With cooling effect and bitter taste, BD is used since ages to treat various ailments. It rejuvenates the whole body enhancing the vigour and vitality upon its routine use justifying its Indian name Punarnava. It has emetic, expectorant, diaphoretic, stomachic, laxative and diuretic properties (Nadkarni, 1976). Due to its multifarious therapeutic potential against abdominal pain, diarrhoea, epilepsy, dysentery, urinary and kidney complications, jaundice, anemia, pneumonia, splenomegaly, etc., it is extensively used globally as a rejuvenator in various medicinal formulations, especially in Asia, Africa and Latin America. It is a well-known tonic, blood purifier and a uterine bleeding preventer to check *postpartum* heamorrhage. Also, it is useful in wound healing and skin ailments like itching and eczema (Agrawal et al., 2011). It improves digestion, maintains healthy body mass index, prevents anaemia, hernia and respiratory distress. Its root extract is a potential kidney, heart and liver stimulant. It protects dilapidated kidneys in the diabetic. Due to its diuretic, renoprotective and laxative properties, it is useful in treating asthma, constipation, cough and detoxifying body. It is reported to be used against dropsy, ascites, gonorrohea, swelling of legs, intestinal worm infestation, jaundice and other hepatic complications. It relieves from inflammation and joint pains, boosts immunity and strengthens the lungs. Its root paste may be used as a miraculous dressing for ulcers and swellings. It is very much beneficial in nerve flaws and paralysis, in treating fever and a loss of appetite (Bhowmik et al., 2012). In case of ascites, liver cirrhosis is normally followed by congestive heart failure. Here herbal diuretics are preferred to correct abnormal fluid dynamics in the body. Having anti-inflammatory property, the whole leaf or its formulation is taken orally or applied locally for wound-healing or to detoxify from scorpion sting and snake bites (Mishra et al., 2014).

### Pharmacological properties of BD

**FIGURE 2:**
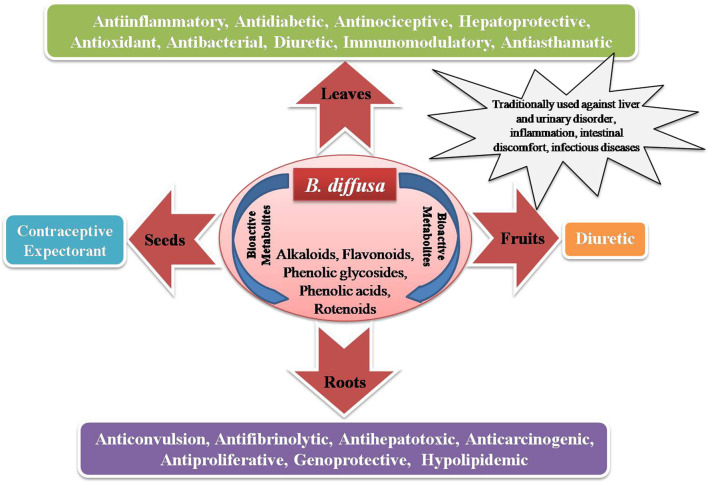
Graphical presentation of the various medicinal virtues of BD.

Different BD parts like root, leaf, flower, fruit and seed are frequently employed to treat various ailments, either on their own or as a bioactive component in medicinal formulations (Rao, 2016). Distinct BD parts have diverse phytochemical compositions with diversified therapeutic benefits. Thoroughly analysed literature from databases agrees that BD and its parts pose numerous bioactive large variety (quality) and amount (quantity) of phytocompounds (Figure 1). The leaves are utilised as a leafy vegetable by Odias in India owing to the superior nutraceutical aspects, high on protein, fatty acid, vitamin C and B complex and calcium. Figure 1 highlights the active substances in BD leaves and the notable bioactivities. Owing to its numerous bioactivities including as antibacterial, antidiabetic, antioxidant, anticancer, hepatoprotective, renoprotective, cardioprotective, diuretic, anti-inflammatory, and immunomodulatory characteristics, in-depth research has been performed on BD (Figure 2).

Several studies conducted globally on this wonder herb correlated the diverse phytocompounds and the bioactive potentials. All the reports are classified under five categories of bioactivity, *i.e.*, metabolic disorders, antimicrobial activities, anti-inflammatory and healing properties, anticancer property and major organ protective role of different compounds extracted from BD of its parts for better understanding and ease of citation. The extracts and isolated phytocompounds of BD studied *in vivo*, *ex vivo* and *in vitro* in the last decade between 2013 and 2023 are compiled and presented in Table 1 through 5 (Table 1: the prophylactic role; Table 2: antibacterial, antihelminthic and antimalarial activities; Table 3: anti-inflammatory, anticataract, wound and gastric ulcer healing properties; Table 4: anticancer and antiproliferative activities; and Table 5: organ protective role).

**TABLE 1:** A list of bioactive properties of BD-extracts to counter metabolic disorders.

Sl. No.	Dosage	Study model	Result	Source
1. Antidiabetic and hypoglycemic property				
1	Methanol, aqueous extract of BD root (200 mg/kg)	STZ-induced diabetic rat	Attributed to or better peripheral glucose uptake, pancreas stimulation or *β*-cell insulin secretion, glucose levels dropped by 9.91% in aqueous and 18.88% in methanol extracted treatments	Malhotra et al. (2014a)
2	Methanol, aqueous extract of BD root	STZ-induced diabetic rat	Brilliant hypoglycaemic and hypolipidemic effect exhibited by both extracts. The total cholesterol (TC), triglyceride (TG), VLDL, LDL significantly decreased, and HDL that could protect pancreas increased. The sd-LDL, lb-LDL, and LDL levels decreased *ex vivo* with maximal effect on sd-LDL	Malhotra et al. (2014b)
3	BD methanol extract (BDME) (100 and 200 mg/kg)	STZ-induced diabetic rat	BDME pretreatment appreciably altered the blood glucose, blood plasma enzymes SGOT, SGPT, and ALP, weight loss, total protein, serum insulin and liver glycogen levels, and restored antioxidant enzyme (SOD, CAT, and GPx) activities	Alam et al. (2018)
4	Ethanol extract of BD leaves (ELBD) (500 mg/kg)	STZ-induced diabetic rat	Restored the elevated renal and hepatic markers and carbohydrate metabolising enzyme levels to normal, like glibenclamide in lowering glucose and lipid (serum cholesterol, triglycerides, VLDL-C, LDL-C and phospholipids) levels, augmenting the level of HDL-C in test rats	Vasundhara and Devi (2018)
5	(Ethyl acetate, ethanol and water) extracts from the aerial parts of BD (100–250 μg/mL)	Sprague Dawley rat	Dose-dependent, it inhibited jejunal glucose and enhanced muscle glucose uptake; exhibited strong antioxidant potentials and high anti-lipidemic and anti-hyperglycemic activities by inhibiting fat and carbohydrate digestion besides abdominal glucose intake and increasing muscle glucose uptake	Oyebode et al. (2018)
6	Methanol extract of leaves	STZ-induced diabetic rat	Reduced glucose and HbA1c and elevated serum insulin and insulin receptor A after 28-day treatment at par with glibenclamide. Both standard drug and the extract modulated carbohydrate-metabolising enzyme and liver glycogen activities significantly by up-regulating mRNA expression of Glucose Transporter-2 (Glut2)	Ibrahima et al. (2018)
7	Methanolic root extract, purified bioactive fraction	STZ-induced diabetic rat	Significant reduction in fasting blood glucose (FBG) and glycated haemoglobin (HbA1c) after 14-week treatment; superior plasma lipid profile, free fatty acid levels, phospholipids, HMG-CoA reductase activity, conjugated diene levels, lipid hydroperoxide levels and malondialdehyde (MDA); antioxidant enzyme (SOD, CAT, GPx, GR, and GST) activity was restored	Akhter et al. (2019)
8	Methanolic root extracts of *W. somnifera*, BD and combination (200 mg/kg/day)	Dexamethasone-induced diabetic rat	Both extracts reduced blood glucose level, serum TG, TC, VLDL levels, and increased HDL levels; while there was no change individually, combined it increased body weight and significantly decreased the LDL levels in diabetic rats	Mini et al. (2020)
9	Root, stem and leaf methanol extract of di-, tetra- & octa-ploid BD species	Alloxan-induced diabetic Swiss albino male rat	Diploid cytotype showed maximum activity against diabetes in alloxan-induced diabetic rats compared to tetra- and octa-ploid cytotypes; phytoconstituents concentration dropped with increasing ploidy levels	Sharma et al. (2021)
2. Hypolipidemic property				
1	70% ethanol root extract	Oxidised cholesterol -induced hyper-cholesteremic rat	TC (34.39%), TG (42.13%), and LDL (48.30%) levels significantly decreased; CAT (42.45%), SOD (27.32%), and GR (15.62%) increased in 4-week treated compared to hypercholesterolemic rat; antioxidant activity improved by 19.97%	Singh et al. (2014)
2	Whole plant extract 150 mg/kg treated for 45 days	Isoproterenol (85 mg/kg, I*.*P.) -induced myocardial infarction	TG, TC, FFA, LDL, VLDL increased and HDL level in serum and heart significantly decreased; biochemical parameters in the treated groups were comparable to control	Shameela et al. (2015)
3. Antiobesity property				
1	n-hexane, n-butanol, chloroform and water methanol extracted fractions (200 mg/kg/d)	Sprague–Dawley (SD) rats	Water-fraction of extract (200 mg/kg/d) lowered the body weight and reduced the relative fat pad weight significantly compared to high-fat diet fed animals; Hx-F, Chl-F, and n-But-F did not alter body weight or fat pad weight	Khalid et al. (2022)

**TABLE 2:** A list of the reported antimicrobial and antiparasitic activities of BD.

Sl. No.	Dose	Microbe and parasite	Salient observation	Source
1. Antibacterial property				
1	Methanol and aqueous root extract	*B*. *subtilis*, *S*. *aureus*, *E*. *coli*, *K*. *pneumonia* and *P. aeruginosa*	Methanolic extract was more effective than aqueous, significantly more so on Gram-positive bacteria than Gram-negative. Methanolic extract was more effective (17.5 mm) against *S. aureus* and least (8.5 mm) against *E. coli*	Malhotra et al. (2013)
2	Aqueous extracts of leaf, stem and root	Bacterial human pathogens	Bacterial sensitivity observed as *S. typhi>S. aureus>E. coli* in that order. Plant parts exhibiting antibacterial activity in the order of root>leaf>stem	Majgaine and Verma (2017)
3	Methanol extracts of di-, tetra- and octaploid BD root, stem and leaf	*B. subtilis*, *S*. *aureus*, *E coli*, *K, pneumoniae*, *P aeruginosa*	Diploid cytotype extract had maximum (27.7 mm) zone of inhibition against *S. aureus* followed by *E. coli* (26.0 mm); minimum ZOI detected against *P. aeruginosa* (2.0 mm) at 80 μL concentration	Sharma et al. (2021)
4	BD extract	*M*. *tuberculosis* H37Rv infection *in vitro* in RAW 264.7, A549, NuLi-1 and BEAS-2B	Impedes pro-inflammatory cytokine production, NO release and regulates immunomodulatory mediators in *Mtb*-infected RAW 264.7 and BEAS-2B cells; significantly (*p* < 0.05) reduced cell viability in RAW 264.7 and A549 in a dose-dependent manner; no significant reduction in viability when treated with 2.5–20 mg/L concentration in BEAS-2B, NuLi-1 cells and splenocytes proving therapeutic role in *Mtb*-infection	Zheng et al. (2021)
5	Ethanol extract of leaf	*P. aeruginosa*	Exhibited significant antibacterial and anti-biofilm activity against *P. aeruginosa*; further confirmed through computational analyses and docking	Shravani et al. (2023)
2. Antimalarial property				
1	Crude methanol extract of root	*Plasmodium berghei NK 65*	Suppressive, curative and prophylactic potential against malaria (*Plasmodium berghei* NK 65), a chloroquine resistant strain	Adefokun et al. (2015)
3. Antileishmanial property				
1	100 mg/kg BD and 400 mg/kg *Ocimum sanctum* (OS), 5 days	*Leishmania donovani* infected BALB/c mice	Maximum parasite clearance from the infected with combined therapy; cell-mediated immunity was upregulated in extract-treated groups with increased delayed-type hypersensitivity responses and enhanced immunoglobulin G2a (IgG2a) levels	Kaur et al. (2015)
4. Antihelminthic property				
1	Ethyl acetate, methanol and aqueous extract of root	*Pheritima posthuma*	Antihelmintic activity by all extracts at 10, 25 and 50 mg/mL doses in a dose-dependent manner; activity was maximum by methanol-extract, followed by ethyl acetate and aqueous extracts	Tiwari et al. (2022)

**TABLE 3:** A list of reported anti-inflammatory and curing properties of BD.

Sl. No.	Dose	Model employed	Salient observation	Source
1. Anti-inflammatory and analgesic properties				
1	Punarnavasava ayurvedic liquid	Rat	Inhibited carrageenan-induced paw edema, cotton pellet-induced granuloma, formalin-induced pain, and pyloric ligation-induced ulceration	Gharate and Kasture (2013)
2	Pet. ether, dichlor-methane, ethanol and water extract (200 mg/kg b.w.)	Rat	Ethanol extract was most potent against formalin mediated inflammation and acetic acid mediated pain as an analgesic	Shubha and Govindaraju (2013)
3	Pet ether extract of root 500 and 1,000 mg/kg p.o	Freunds complete Adjuvant -induced arthritic rat	Sub-plenter adjuvant administration significantly reduced paw swelling after; paw edema inhibited by 500 mg/kg (32.31%) and 1,000 mg/kg (62.88%) extracts by the 21st day, while Indomethacin treatment showed maximum 77.07% inhibition by the 21st day	Dapurkar et al. (2013)
4	Aqueous leaves extracts (200 and 400 mg/kg)	Rat	Extract preadministration showed dose-dependent anti-inflammatory activity against sub-acute and acute inflammation in cotton-pellet mediated granuloma and carrageenan-prompted paw edema, probably by inhibiting the chemical mediators	Sudhamadhuri and Kalasker (2014)
5	Rotenoid rich fraction and phophatidylcholin soluble fraction	Female Sprague –Dawley rats	RRF-PC had 64% anti-inflammatory activity *in vivo* in 5 h compared to 48% of RRF and 50% of ibuprofen. Plasma concentration of boeravinone B was two times higher in RRF-PC (75 ng/mL) compared to RRF (40 ng/mL)	Bairwa and Jachak (2015)
2. Antiarthritis property				
1	Petroleum ether, chloroform, methanol, water based root extract	FCA -induced arthritic rat	Methanol extract was more effective; paw volume was reduced and body weight improved in treated arthritic rat with more paw volume, less body weight, increased WBC and erythrocyte sedimentation rates, and decreased RBC and haemoglobin; haematologically stabilised with maximal effects at 400 mg/kg	Parmar et al. (2018)
3. Wound healing				
1	Methanol (ME) and chloroform (CE) based leaf extract	Mice	ME significantly increased viability and migration of human keratinocyte cells compared to untreated or CE-treated cells; topically applied ME reduced wound area significantly (91%) by the 14th day compared to control (22%); secondary metabolites D-pinitol and caffeic acid facilitated the healing	Juneja et al. (2020)
4. Antiulcerative and antacid properties				
1	Punarnavasava, an ayurvedic formulation	Rat	Significantly reduced piloric ligation-induced gastric juice secretion, free acidity as well as total acidity and ulcer index; pronounced effect on all the parameters compared to Ranitidine	Gharate and Kasture (2013)
5. Anticataract property				
1	Alcoholic root extract, 100, 200, 400 mg/kg; 28-day	Rat	Delayed cataractogenesis in galactose-induced cataract with reduced galactitol level and aldose reductase activity in isolated rat eye lens	Lakshmi et al. (2017)

**TABLE 4:** A list of reported anticancer and antiproliferative properties of BD.

Sl. No.	Dose	Model employed	Salient observation	Source
1	Punarnavine (15 mg/kg bw/d)	Human umbilical vascular endothelial cells (HUVECs) Ehrlich ascites carcinoma tumor model in Swiss female albino mice	Suppressed HUVEC proliferation; considerably reduced endothelial cell migration and invasion, and capillary structure formation of HUVEC, downregulated VEGF-A; 50 mL Punarnavine significantly inhibited MMP-2 & MMP-9 expression *in vitro*; 15 mg/kg bw/d Punarnavine treatment decreased ascitic fluid (60.94%) & tumor (86.40%) volume in Ehrlich ascites model; reduced peritoneal angiogenesis exhibited anti-angiogenic property of Punarnavine	Saraswati et al. (2013)
2	Ethanol based leaf extract (250 and 500 mg/kg)	Swiss albino mice	Anticancer role against Dalton’s ascitic lymphoma (DAL) cell-induced carcinogenesis was proved with 18.40 ± 1.41 days survival of control (DAL-infected tumour) group and 31.12 ± 1.0 (41%) and 33.12 ± 0.9 (46.23%) days for 250 and 500 mg/kg treated EBD groups; average tumour volume in DAL-treated was 2.68 ± 0.11. EBD-treated group had 1.36 ± 0.10 (250 mg/kg) and 0.98 ± 0.04 (500 mg/kg); total WBC count, protein and PCV increased, haemoglobin and RBC counts decreased in DAL-infected, which were normal in EBD	Kayande and Kushwah (2014)
3	Methanolic leaf extract (25, 50, 100, and 200 μg/mL)	MCF-7 cell	Dose-wise, the cytotoxicity was 13.9%, 27.96%, 43.65%, and 52.86. IC_50_ of 69.18 μg/mL and cell viability of 47.14% were observed at 200 μg/mL	Muthulingam and Chaithanya (2018)
4	Methanolic (ME) and aqueous (AE) extracts	KB oral cancer cell line	Significant cytotoxicity with IC_50_ value between 36 and 30 μg/mL; ME exhibited better apoptotic DNA fragmentation and apoptotic inducing potential compared to AE	Gunaseelan et al. (2022)
5	BD extract at 800 μg/mL	MCF-7	Test sample at 800 μg/mL concentration exhibited cytotoxicity of about 65.1 ± 1.2 in 48 hr-incubated MCF-7 breast cell-line	Remya et al. (2018)

**TABLE 5:** A list of the reported protective role of major human organs by BD.

Sl. No.	Dose	Model used	Salient observation	Source
1. Cardioprotective property				
1	Ethanol extract (BD whole plant)	As_2_O_3_ toxicity H9c2 myoblasts	Reduced lactate dehydrogenase (LDH) levels, oxidative stress and calcium influx	Vineetha et al. (2013)
2	Ethanol leaf extract	Ang II-induced hypertrophy in H9c2 cardiomyoblasts	Elevated intracellular ROS levels and surplus creation of mitochondrial superoxide radicals in hypertrophied cells indicated development of oxidative stress during hypertrophy; significantly prevented increase of NADPH oxidase, xanthin oxidase and reduction of aconitase, thioredoxin reductase in cells exposed to Angiotensin II (Ang II) and hypertrophied cells	Prathapan et al. (2013); Prathapan et al. (2014)
3	Ethanol extract (BD whole plant)	Doxorubicin-induced myocardial toxicity in rats	Improved doxorubicin-mediated cardiotoxicity by reducing the elevated levels of cardiac markers, *e.g.*, creatine kinase, LDH and other markers, *e.g.*, ALT, AST, ALP; uplifted the reduced levels of antioxidant enzymes (SOD, CAT, and GR)	Nimbal and Koti (2016)
4	Polyphenol-rich ethanol extract	Angiotensin-II-induced cardiac hypertrophy and fibrosis in rats	Significantly reduced fibrosis in heart and protected it from pathological hypertrophy and cardiac anomalies, probably by reducing oxidative stress	Prathapan et al. (2017)
2. Hepatoprotective property				
1	Methanol leaf extract (200 mg/kg)	Rifampicin mediated liver toxicity in rat	Increased ALT, AST, ALP, GGT, LDH and bilirubin levels and decreased protein levels in serum; restored liver functioning to normal level	Muthulingam (2014)
2	Eupalitin-3-O-*b*-D-galactopyranoside from leaf	CCl_4_ (0.1%) induced cytotoxicity in HepG2	Protected HepG2 cells ranging from 62.62% (500 μg/mL) to 70.23% (1,000 μg/mL) against CCl_4_ (0.1%) induced hepatotoxicity	Thajudeen et al. (2022a)
3	Boeravinone B (200 μg/mL) and caffeic acid (200 μg/mL)	Galactosamine (40 mM) induced cell toxicity in HepG2	The hepatoprotection was comparable to the effects of regular sylimarin	Thajudeen et al. (2022b)
4	Ethanolic leaf extract (250 mg/kg b.w./d)	Cr^6+^-induced nephrotoxicity in *Ctenopharyngodon idellus*	Metal exposure elevated chromium accumulation and malondialdehyde (MDA) level, altered kidney tissue architecture, elevated kidney function markers and reduced antioxidant enzyme (superoxide dismutase, catalase and glutathione-S-transferase) activities. The treatment improved tissue architecture, and brought the level of kidney markers, antioxidants and genes expressions to near normal	Handa and Jindal (2023)
3. Renoprotective and dieuretic properties				
1	BD root extract and standard drug Enalapril	Rat	Lowered both systolic and diastolic blood pressure by 30th day; serum Cr, BUN, phosphorus, urine protein, ALP and GGT significantly reduced by 60th day; neither group’s nephrosonographic image showed any sign of further improvement even after the 90th day	Oburai et al. (2015)
2	Ethanolic root extract	Cisplatin-induced nephrotoxicity in rat	Decreased apoptotic and necrotic cells, and attenuated ROS synthesis; nephroprotection by phytochemicals like polyphenols (4.5 ± 0.02), flavonoids (4.2 ± 0.08) and tannins (6.5 ± 0.3) in mg/g present in extract	Kalaivani et al. (2015)
3	Hydroalcoholic root extract (50, 100 and 200 mg/kg)	Cisplatin-induced acute kidney injury in Wistar rat	200 mg/kg b.w. in substantial nephrotoxicity with significantly elevated serum creatinine and blood urea, decreased concentrations of reduced glutathione and superoxide dismutase, elevated TNF-a level in renal tissues in Wistar rats significantly increased serum creatinine, blood urea N, reduced oxidative stress and inflammatory markers; displayed antiapoptosis in kidneys by reducing caspase-3 expression	Karwasra et al. (2016)
4	Ethanol root extract, *Rheum emodi*, flowers of *Nelumbo nucifera* and stem bark of *Crataeva nurvala* (150–300 mg/kg)	methotrexate-induced nephrotoxicity in Wistar rats	Extract containing 27% phenols and 15% flavonoids exhibited 75% DPPH scavenging potential; IC_50_ of the selected best combination to scavenge DPPH and inhibit xanthine oxidase were 80 and 74 mg/mL; combined treatment enhanced histological parameters, oxidative stress markers and kidney function markers in methotrexate-induced nephrotoxic rats significantly	Sharma et al. (2020)
5	BD–*Tinospora cordifolia* (TC) Combination	Diclofenac (DCF)-induced nephrotoxicity	Due to polyphenols’ nephroprotective property via gene modulation in oxidative stress, inflammation, renin-angiotensin system (RAS) and other pathways, the mixture lowered renal oxidative and inflammatory stress and substantially improved nephroprotection	Gaurav et al. (2022)
4. Effect on female reproductive organ				
1	BD extract (100 mg/kg)	Female rat	A significant dose-dependent drop in ovarian weight was observed in the extract-treated group with tissue samples displaying normal histology; group receiving 100 mg/kg extract had abnormal oestrous cycle producing no litter. Thus BD had antifertility effect	Adebajo et al. (2018)
2	Hydroalcoholic root extract	Mice, rat	Extraneous application of noradrenaline reduced prostate weight and contractile response of prostate gland and vas deferens; histoarchitecture of prostate gland improved compared to model rat	Vyas et al. (2013)

### Protective role against metabolic disorders

Globally, 422 million people suffered from diabetes as per the global reports on diabetes by the World Health Organisation (WHO), 2023. Three out of every four diabetic belonged to younger age groups primarily in the under-developed and developing economies greatly impairing their productivity and productive years of life. Nearly 1.5 million diabetic die each year due to hyperglycaemia related complications. The diabetic is also prone to infectious diseases like TB (tuberculosis), malaria and HIV/AIDS. As the cases are rising alarmingly, diabetes is becoming a global challenge. With increasing number of diabetics in the present era majorly due to sedentary lifestyle, anomalous food habits, stress, etc., India is becoming the “diabetic hub”.

**FIGURE 3:**
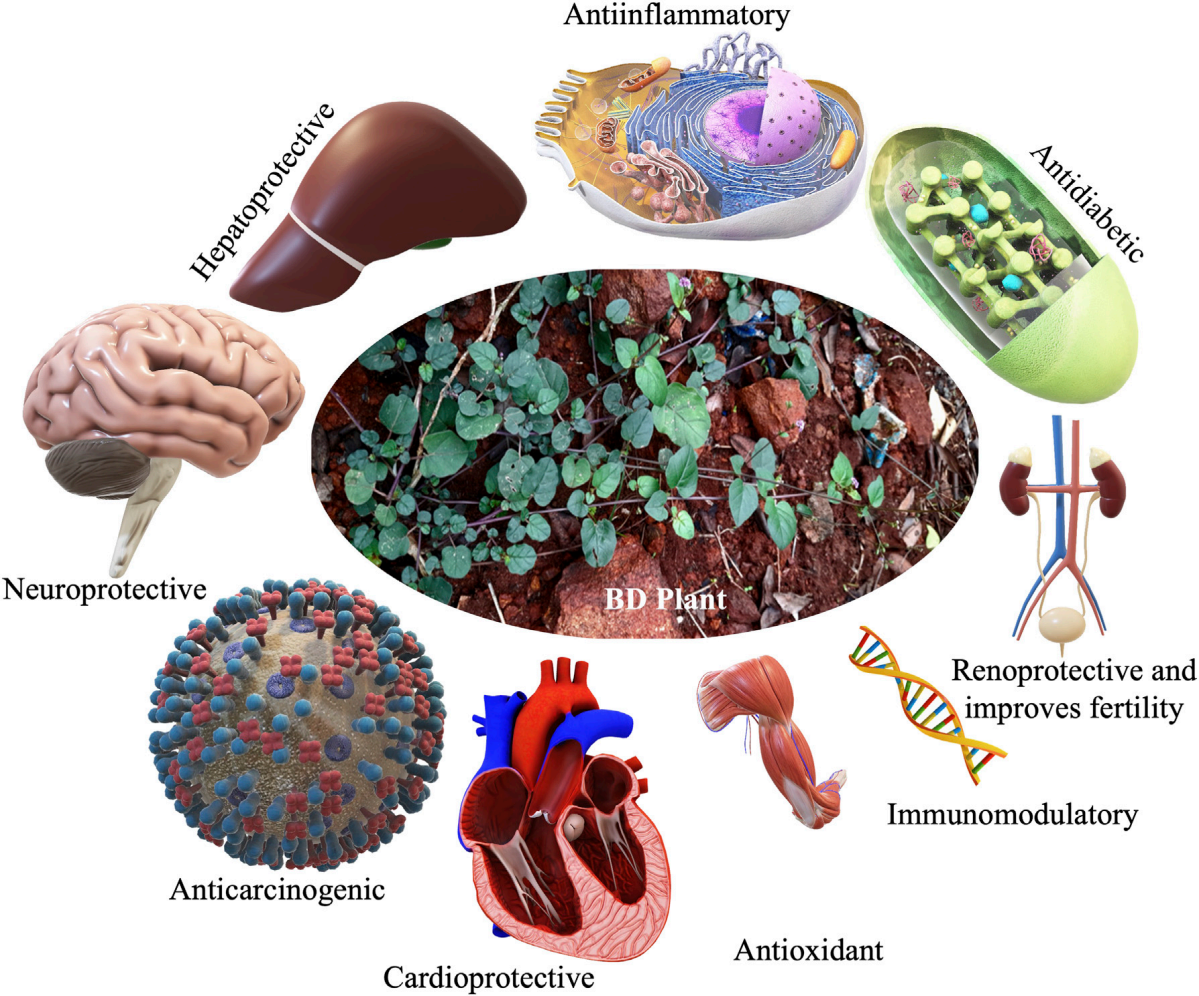
A schematic presentation of the various medicinal benefits of BD on human tissues and organs.

The compared antidiabetic potential of root, stem and leaf extracts of BD to standard over-the-counter drugs in streptozotocin-induced diabetic animal models significantly reduced blood glucose level, downregulated oxidative stress markers and showed free radicals scavenging ability (Montefusco-Pereira et al., 2013; Alam et al., 2018; Vasundhara and Devi, 2018). Ethanol-based leaf-extract (500 mg/kg, 45 days) altered carbohydrate metabolising enzymes considerably to near normal in Streptozotocin (STZ) (60 mg/kg I.P.) mediated diabetic rat (Vasundhara and Devi, 2018), validating its antidiabetic effect. Significantly reduced liver glycogen content in diabetic rat was restored with leaf-extract treatment, mediated through inhibited jejunal glucose uptake and augmented glucose uptake by skeletal muscle (Oyebode et al., 2018). Increased liver enzymes (ALP, SGPT, and SGOT) levels in plasma upon being released from liver cytosol into the bloodstream in STZ-induced diabetic rat confirmed liver necrosis. The methanol extract completely reversed this clinical condition in test rats. Elevated glucose levels in diabetic rats due to oxidative stress that inactivated antioxidant enzymes like SOD, CAT, and GPx possibly through glycation was stabilised by administering methanol-based BD extract (Alam et al., 2018). Elevated renal and hepatic marker levels in STZ-induced diabetic rat was normalised after treating with ethanol-based leaf-extract of BD (Vasundhara and Devi, 2018). Most of the therapeutic manifestations of BD are attributed to its incredible antioxidant properties (Das et al., 2022). Metabolic disorders often increase due to an imbalance in oxidative status. A compilation of the prophylactic role of BD reported in the last decade is presented in Table 1. The reported possible modes of action of it as an antidiabetic as proposed by various researchers are compiled in Figure 3.

### Antimicrobial activities of BD

The extracts of root, stem and leaf parts of BD have demonstrated their antimicrobial properties. Higher (200 and 100 mg/mL) doses of root extract inhibited *S*. *aureus* better with a 12–16 mm zone of inhibition, whereas the leaf extract showed similar result of 11–13 mm zone of inhibition even at lower (50 and 25 mg/mL) doses. While root extract was effective against *S*. *typhi* also, none was effective against *E*. *coli* (Majgaine and Verma, 2017), contradicting the findings of few other researchers. The antiplasmodial effectiveness was examined on *Plasmodium berghei* NK 65, a chloroquine-resistant strain, using suppressive, curative and preventive malaria male and female albino mice models. The extract exhibited the best antipyretic effect at 125 mg/kg by the third day in suppressive models confirming its antimalarial activity. The extract reduced the plasma Ca^2+^ levels better at 500 mg/kg than the nifedipine (positive control) treated at 1.043 vs*.* 1.35 mmol/L. Its efficacy in reducing malaria-induced pyrexia by blocking Ca^2+^ channel in the erythrocytes validates its ethnic use as an antimalarial (Adefokun et al., 2015). The evaluated antileishmanial activity of BD and OS on *L*. *donovani* infected BALB/c mice model had the maximum parasite clearance at 5-day BD:OS:100:400 mg/kg combination therapy. It also upregulated cell-mediated immunity with increased delayed hypersensitivity and enhanced immunoglobulin G2a (IgG2a) production. The extract normalised abnormally high serum urea, blood urea nitrogen (BUN), creatinine, glutamic oxaloacetate transaminase and glutamic pyruvate transaminase levels (Kaur et al., 2015). Reports on antibacterial, antihelminthic and antimalarial activities of BD published between 2013 and 2023 are compiled and presented in Table 2.

### Anti-inflammatory, anti-cataract and wound-healing properties of BD

**FIGURE 4:**
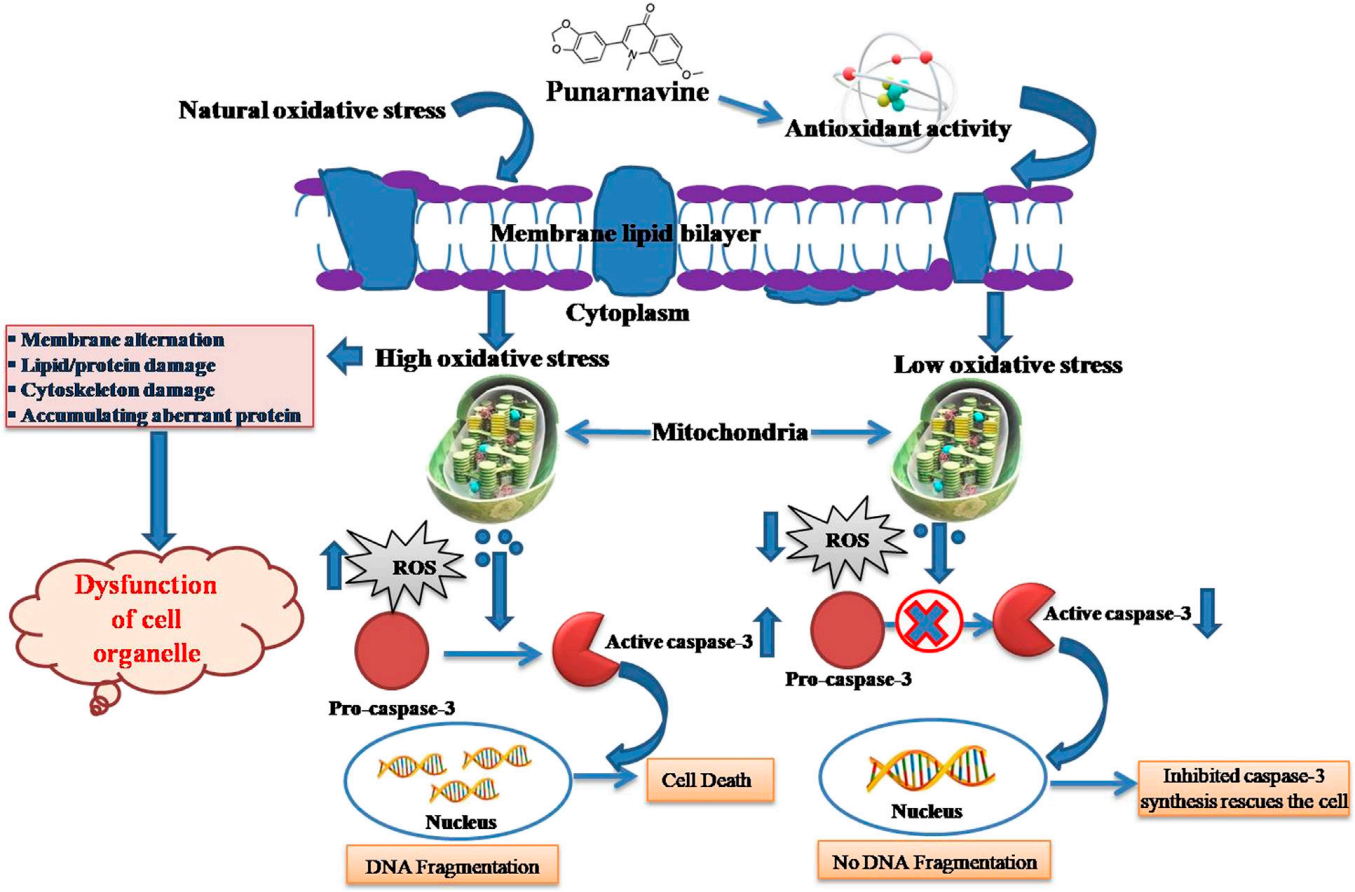
The DNA damage mechanism due to oxidative stress and the role of boeravin in its repair.

Methanol extract of BD leaf reportedly had promising antioxidant and anti-inflammatory activity due to high polyphenolics content. Inflammatory mediators like leukotrienes (LTs) are generated in body via 5-LOX pathway. Polyphenolics, flavonoids, saponin, etc. As antioxidants reportedly exhibited antilipoxygenase activities possibly by blocking arachidonic acid pathway and serving as free radicals scavengers (Ibrahima et al., 2018). BD is reported to have significant anti-inflammatory, anticataract, wound and gastric ulcer healing traits (Table 3). Antioxidant defense mechanism by reducing ROS production and inflammation is schematically presented in Figure 4.

### Anticancer and anti-proliferative properties of BD

The oncoprotective and chemopreventive properties of phytocompounds from BD are reported by many, often due to the antioxidant properties. Alcohol and water-based extracts could contain several biocompounds like punarnavine, punarnavosides, rotenoids, reducing sugars, starch, and lignans, liriodendrin, syrigaresinol and several boeravinones (boeravinone A–J) (Das et al., 2022). Boeravinones G and H reportedly inhibited drug efflux potency of BCRP/ABCG2, the breast cancer resistance protein, a multidrug transporter that induces chemotherapy resistance in cancer cell (Ahmed-Belkacem et al., 2007). As BD reportedly controls unregulated cell growth through S phase inhibition and apoptosis, its cytotoxic activity was effective against many tested cancer cell lines proving its claimed anticancer activity. Relevant reports published between 2013 and 2023 are presented in Table 4.

### Organ protective role of BD

**FIGURE 5:**
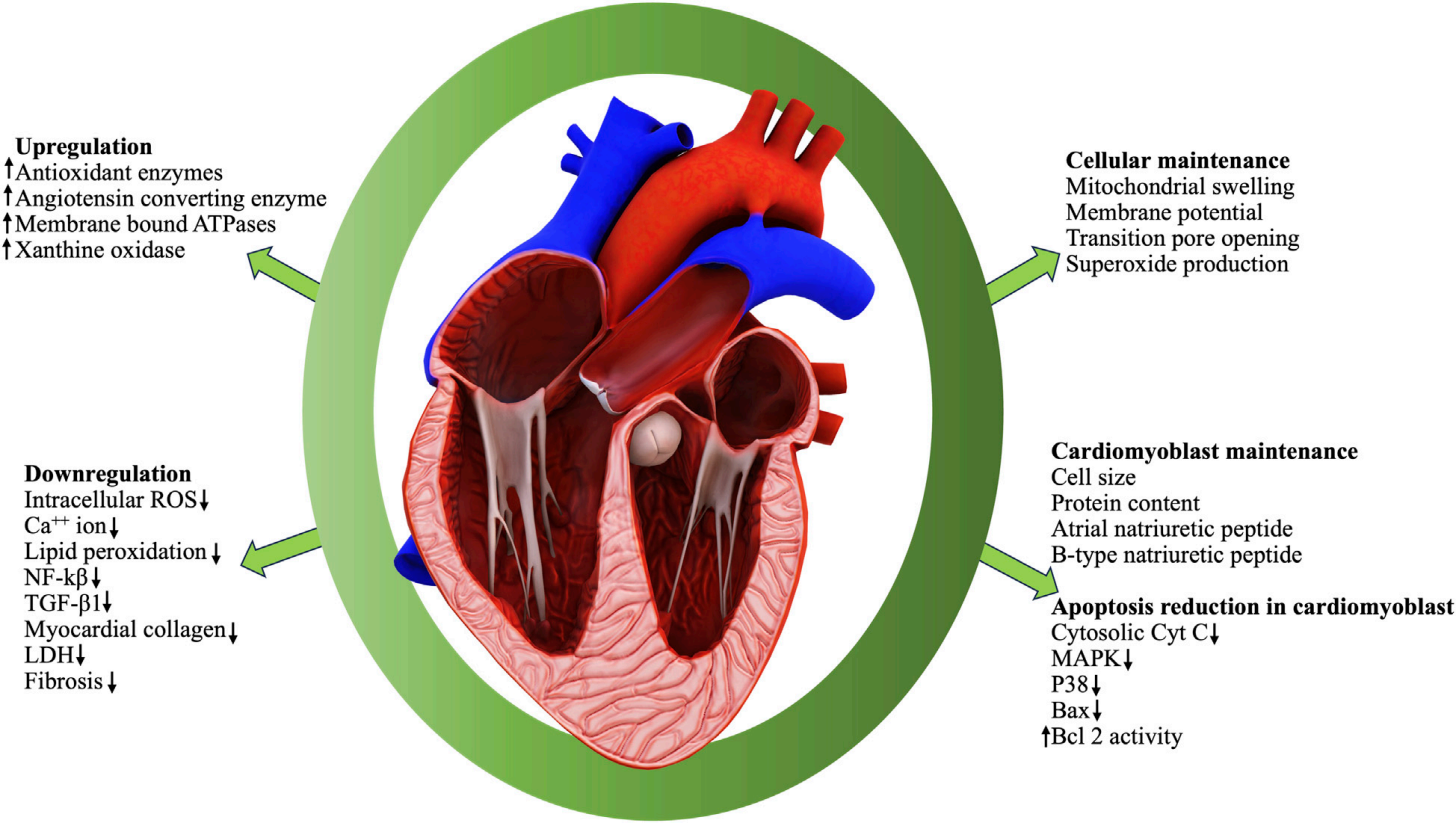
Cardioprotective role of bioactive compounds in BD.

The whole BD is popular among ethnic community for its excellent healing properties against diseases which is time-and-again validated through scientific investigations for the proclaimed ethnopharmacological properties. Vital organs of human body such as heart (Figure 5), liver and kidney are protected by the phytocomponents safeguarding them. The various reports pertinent to the major organ protective roles of BD published in the last decade are compiled and presented in Table 5.

### Bioinformatics approaches to decode the benefits

Crude BD extract is highly effective and traditionally used against mastitis caused by *S. aureus*. The antimastitis activity of the phytochemicals in BD was examined *in silico* by Sruthy et al. (2018) through docking analysis. Based on ADMET properties, 20 BD phytochemicals were selected for docking. Boeravinone A, B, C, D, E, and F had good docking scores and interacted better with the active sites of the target receptor protein of *S. aureus*. This study helped design novel drugs to treat mastitis. Similarly, Kaviya et al. (2022) analysed the hot and cold solvent extracted BD phytochemicals from leaves, stems and roots, and found that ethanolic root extract demonstrated clear zones of inhibition for *P. aeruginosa* (8 mm) and *S. aureus* (20 mm) at 200 µg concentration. Molecular docking of 2-(1,2 dihydroxyethyl)-5-{[2,5,7,8-tetramethyl-2-(4,8,12-trimethyltridecyl)-3,4-dihydrochromen-6-yl]oxy}oxolane-3,4-diol exhibited minimal binding score against *P. aeruginosa*. Patients with nephrotic syndrome are resistant to steroid and immunosuppressive treatments. Many natural compounds are traditionally used to treat chronic diseases. Sahu et al. (2022a) screened the potency of 66 bioactive compounds phytochemicals in BD against mutant nephrin protein, against wild and mutant Ig4 domain of nephrin protein through AutoDock raccoon. Simulated molecular dynamics in 100 ns trajectory of Ig4 domain of nephrin protein with Boeravinone M and Boeravinone E showed that the latter’s binding increased the compactness, effectively modulating the stability and function. Sahu et al. (2022b) extensively investigated the anticancer properties of BD phytochemicals Boeravinone A, B, C, D, E, F, G, H, I, and J. While analysing binding efficacy and free energy landscape of both the native and mutant CDK2AP1 protein with these by molecular docking, Boeravinone J showed the best binding affinity (−7.9 kcal/mol) towards native CDKAP1. MD simulation for 100  ns suggested that H23R mutant-Boeravinone J complex had the minimum structural changes with less conformational mobility compared to C105A mutant. Such study can help develop Boeravinone E therapeutics.

The *in vitro* molecular docking analysis by JunaBeegum et al. (2017) to determine the potent inhibitory effect of recombinant NS3 genotype 3b catalytic activity of ethanolic extract of BD found that most flavonoids were deeply bound in HCV NS3 protease and interacted with the catalytic triad. The lead molecules liriodendrin, 3,5,6-tri-hydroxyl-4-methoxy flavone7-β-D glucoside, astragalin, caffeoyl tartaric acid, quercetin, 3,3,5,7-tetra-hydroxyl-4-methoxy flavones, boerhavin A, 4,25-secoobscurinervan and 3,5,7,2,5-penta-hydroxyl flavones had anti HCV activity. With −10.72 kcal/mol energy score, liriodendrin was the best interacting compound. Especially, the flavonoids and triterpenoids phytochemicals direct inhibited HCV NS3 protease. They found BD as a potential natural non-toxic anti-HCV agent that inhibited NS3 protease and reduced viral counts inside hepatocytes.

The severe acute respiratory syndrome coronavirus 2 (SARS-CoV-2) responsible for the ongoing COVID-19 pandemic affected over 110 million people worldwide (https://covid19.who.int/), with no clearly targeted efficient therapeutic yet. Finding a suitable docking ligand to inhibit the function of the responsible protein groups is continuously being researched (Abdalla et al., 2021; Mohapatra et al., 2021a; Mohapatra et al., 2021b; Mohapatra et al., 2022; Mohapatra et al., 2023). 3CL-like protease (3CL^pro^) is a non-structural protein involved in post-translational modification in SARS-CoV-2, and is vital to viral replication (https://www.news-medical.net/news/20210328/Boerhavia-diffusa-possess-potential-therapeutic-properties-against-COVID-19.aspx). Thus, 3CL^pro^ could be a preferred drug target.

Surya and Praveen (2021) examined 2-3-4 β-Ecdysone, Bioquercetin, Boeravinone J, Biorobin, Boerhavisterol, Liriodendrin, Kaempferol, Quercetin and Trans-caftaric acid through molecular docking as the major active BD phytochemicals. Biorobin (−8.17 kcal/mol), Bioquercetin (−7.97 kcal/mol) and Boerhavisterol (−6.77 kcal/mol) had low binding energies as ligands against SAR-CoV-2 main protease, and favoured efficient docking and protease inhibition. The drug-likeness property of Bioquercetin, Biorobin, and Boeravisterol was ADME analysed, and Boeravisterol was the most suitable that obeyed the Lipinsky’s rule. Comparative docking of these carried out also against MERS Mpro showed that the obtained binding energies were unfavourable.

### Toxicity studies and clinical trials

Because of the age-old tried and tested use with no known side-effect, traditional herbs and their products are often considered as safe as ethnomedicines. However, few toxicity studies on BD extracts have been reported to empirically verify and validate this perception. Karwasra et al. (2016) observed no adverse effect level in BD root extract even at 1,000 mg/kg in Wistar rats. Orisakwe et al. (2003) studied the acute and subchronic toxicity of BD leaf at 500, 1,000, and 2,000 mg/kg aqueous extract in albino mice and rats, and the lethal dose (LD_50_) for both was more than 2,000 mg/kg (p.o.). With increased food and fluid intake, the rats treated with the extract had a progressive body weight gain, significantly higher than control. Also, there was no significantly comparable change in absolute and relative weights of the vital organs, proving its safety and efficacy.

Adults and children suffering from helminth infection became worm-free within 5 days of oral administration of dried BD root powder (Singh and Udupa, 1972). A clinical trial of BD extract as a chemotherapy adjuvant conducted on 50 patients freshly diagnosed as having pulmonary tuberculosis recovered faster than the control. 80% of BD-treated patients were relieved from cough in 4 weeks as compared to 52% from among the control. Likewise, 88% of the patients were afebrile in 6 weeks as compared to 60% among the control, with the similar afebrile state setting in by the 8th week in the control. The BD treated group had a higher average weight gain, and a considerably faster sputum conversion rate (Kant et al., 2001). Aqueous plant extract could significantly reduce osmotic fragility of erythrocyte in polycystic end-stage renal disease (ESRD) by altering the composition of erythrocyte membrane or affecting the intracellular sodium and improving the oxidative stress (Sathyapriya et al., 2009). The haematological, biochemical and urine parameters of patients with Type-I or Type-II diabetic nephropathy (DM) with >500 mg proteinuria daily and >5 mg/dL serum Cr were examined every month for 6 months in a clinical trial after dividing them into two groups (14 patients of 40–70 years with 60.78 ± 6.37 years mean age in group I and 11 of 45–73 years with 60.27 ± 9.00 years mean age in group II). All patients were having edema, anorexia, weakness and vomiting symptoms. While Ramipril (a standard drug) reduced 24-h urine protein excretion significantly, six-month BD treatment decreased it only to a statistically insignificant level (Singh et al., 2010).

## Discussion

By categorising the collected relevant literature and conducting bibliometric analyses, it was observed that the maximum number of literature (15) were related to organ protective role of BD, followed by the literature related to metabolic disorders (12), anti-inflammatory property (9), antimicrobial and antiparasitic (8) and anticancer (5) potentials. This provides a strong reason to consider the plant and it extracts as a panacea to treat numerous physiological, pathological and lifestyle related illnesses.

Numerous crude extraction, purification and optimisation techniques for scale-up have been suggested in recent reports. Chromatographic technique is advantageous in evaluating retinoid and phenolic acids due to the ease of sample preparation, chemicals optimisation and the scope to simultaneously compare several samples. Chromatogram comparison allows detect similarities and differences between the various bioactive extracts under investigation even at the minutest level. Eupalitin-3-O-D-galactopyranoside has powerful hepatoprotective properties against carbon tetrachloride-induced toxicity. It could be crystallised from sedimented residue, making it a simple, quick and the most economical commercially viable scale-up technique. This novel approach was used to isolate Eupalitin-3-*O*-*β*-*D*-galactopyranoside with up to 0.1578% *w*/*w* yield. Obtaining Eupalitin-3-*O*-*β*-*D*-galactopyranoside from hydro-alcoholic extract of BD employing high-performance thin layer chromatography (HPTLC) technique through a single solvent system (toluene:acetone:water:5:15:1) was optimised, validated and quantified.

Due to altered lifestyle, less physical exercise, work stress, increasing physical stress, junk food preference and rising childhood obesity, the number of diabetics is in the rise at an alarming rate in recent time worldwide including India. To address such metabolic disorders, nutraceuticals including herbal therapeutants like BD as dietary supplement could be suggested. BD reportedly has excellent antidiabetic activity that regulates and optimises biochemical factors in diabetic test models as listed in Table 1. Such wonder manifestation of BD is attributable to a probable mode of action of the phytoconstituents to ameliorate diabetes as proposed in Figure 3. More focused clinical trials and evidence-based intensive investigations to validate its efficacy are needed. Further, the multifarious other benefits extended by BD makes it a genuinely wonder herb that needs it due place in the society for sustainable human wellbeing.

Although several studies validate the traditional use of BD, but most of them lack in deciphering the mechanism of action of the underlying bioactive therapeutic phytocompounds in preventing or protecting against infectious diseases. Investigations confirming its molecular-level action against various diseases, the pharmacology and the toxicology of unexplored secondary metabolites if any could be undertaken.

## Conclusion and future prospects

BD reportedly has multiple and multifunctional pharmacological traits including antifungal, anticancer, antibacterial, antidiabetic, antiparasitic, cardioprotective, hepatoprotective, renoprotective, anti-inflammatory and antifertility characteristics as opined and concluded by numerous investigators. Though the reports on the pharmacological properties of BD are many, the molecular action of the therapeutic phytocompounds in preventing or protecting against infectious diseases, for instance, most of them are inconclusive. Although there are multiple reports on antimicrobial activities yet the molecular mechanisms of specific interaction between the purified compounds and the microbial effector proteins/toxins or their interference with molecular pathogenesis are limited. Thus, clinical trials and advance studies targeting the understanding of the mechanisms of action at the molecular level can be further undertaken to decipher it, especially of the isolated unexplored secondary metabolites in BD. Additionally, investigations to understand prophylactic and therapeutic roles of these phytomolecules at the molecular level using appropriate test models need to be prioritised.

## Figures and Tables

**Table:** 

**ABTS**	2,2′-azino-bis(3-ethylbenzothiazolin)
**ALP**	Alkaline phosphatase
**ALT**	Alanine transaminase
**AST**	Aspartate transaminase
**BD**	Boerhaavia diffusa
**BHT**	Butylated hydroxyl toluene
**BUN**	Blood urea nitrogen
**CAT**	Catalase
**Cr**	Creatinine
**DPPH**	2,2-diphenyl-1-picrylhydrazyl
**DW**	Dry weight
**EC50**	Half maximal effective concentration
**EDTA**	Ethylenediaminetetraacetic acid
**FRAP**	Ferric reducing antioxidant power
**GABA**	Gamma amino butyric acid
**GAE**	Gallic acid equivalent
**GPx**	Glutathione peroxidase
**GSH**	Reduced glutathione
**GST**	Glutathione-S-transferase
**IC50**	Half maximal inhibitory concentration
**LDH**	Lactate dehydrogenase
**LOX**	Lipooxygenase
**LPO**	Lipid peroxidation
**LPS**	Lipopolysaccharides
**MDA**	Malondialdehyde
**NK**	Natural killer
**NO**	Nitric oxide
**PBMC**	Peripheral blood mononuclear cell
**PMN**	Polymorphonuclear
**QCE**	Quercetin equivalent
**ROS**	Reactive oxygen species
**SGOT**	Serum glutamic oxaloacetic transaminase
**SGPT**	Serum glutamic pyruvic transaminase
**SOD**	Superoxide dismutase
**STZ**	Streptozotocin
**TBARS**	Thiobarbituric acid reactive substances
**TFC**	Total flavonoid content
**TPC**	Total phenolic content
**TNF**	Tumour necrosis factor
**γ-GT/GGT**	Gamma glutamyl transferase
